# The Role of Statins in Prevention and Treatment of Community Acquired Pneumonia: A Systematic Review and Meta-Analysis

**DOI:** 10.1371/journal.pone.0052929

**Published:** 2013-01-07

**Authors:** Abdur Rahman Khan, Muhammad Riaz, Aref A. Bin Abdulhak, Mohamad A. Al-Tannir, Musa A. Garbati, Patricia J. Erwin, Larry M. Baddour, Imad M. Tleyjeh

**Affiliations:** 1 Department of Internal Medicine, University of Toledo Medical Center, Toledo, Ohio, United States of America; 2 Research and Scientific Publication Center, King Fahad Medical City, Riyadh, Saudi Arabia; 3 Department of Internal Medicine, University of Missouri – Kansas City, Kansas City, Missouri, United States of America; 4 Department of Internal Medicine, King Fahad Medical City, Riyadh, Saudi Arabia; 5 Mayo Medical Library, Mayo Clinic, Rochester, Minnesota, United States of America; 6 Division of Infectious Diseases, Mayo Clinic, Rochester, Minnesota, United States of America; 7 Division of Epidemiology, Mayo Clinic, Rochester, Minnesota, United States of America; 8 College of Medicine, Alfaisal University, Riyadh, Saudi Arabia; BarcelonaUniversity Hospital, Spain

## Abstract

**Background:**

Emerging epidemiological evidence suggests that statins may reduce the risk of community-acquired pneumonia (CAP) and its complications.

**Purpose:**

Performed a systematic review to address the role of statins in the prevention or treatment of CAP.

**Data Source:**

Ovid MEDLINE, Cochrane, EMBASE, ISI Web of Science, and Scopus from inception through December 2011 were searched for randomized clinical trials, cohort and case-control studies.

**Study Selection:**

Two authors independently reviewed studies that examined the role of statins in CAP.

**Data Extraction:**

Data about study characteristics, adjusted effect-estimates and quality characteristics was extracted.

**Data Synthesis:**

Eighteen studies corresponding to 21 effect-estimates (eight and 13 of which addressed the preventive and therapeutic roles of statins, respectively) were included. All studies were of good methodological quality. Random-effects meta-analyses of adjusted effect-estimates were used. Statins were associated with a lower risk of CAP, 0.84 (95% CI, 0.74–0.95), I^2^ = 90.5% and a lower short-term mortality in patients with CAP, 0.68 (95% CI, 0.59–0.78), I^2^ = 75.7%. Meta-regression did not identify sources of heterogeneity. A funnel plot suggested publication bias in the treatment group, which was adjusted by a novel regression method with a resultant effect-estimate of 0.85 (95% CI, 0.77–0.93). Sensitivity analyses using the rule-out approach showed that it is unlikely that the results were due to an unmeasured confounder.

**Conclusions:**

Our meta-analysis reveals a beneficial role of statins for the risk of development and mortality associated with CAP. However, the results constitute very low quality evidence as per the GRADE framework due to observational study design, heterogeneity and publication bias.

## Introduction

The incidence of community-acquired pneumonia (CAP) ranges between 3 and 40 per 1000 inhabitants per year with estimated rates of hospitalization and overall mortality of 40–60% and 10%, respectively [1]. Despite advances in antimicrobial therapy the mortality from CAP has remained relatively constant [2]. CAP has been associated with both short-term (within 30–90 days after CAP) [3] and increased long-term mortality [4]–[5]. In view of its common occurrence, an aging population and rising healthcare costs, CAP presents a major problem and is one of the leading causes of death [6]. Therefore, besides anti-microbial therapy other potential approaches should be considered for a better outcome of CAP.

Several factors have been postulated for the adverse outcomes in CAP including acute lung injury (ALI), vascular dysfunction and coagulopathy due to a dysregulated inflammatory response caused by invading microorganisms. The pathogenesis of ALI and acute respiratory distress syndrome (ARDS) includes a ‘cytokine storm’, which is involved in the initiation and amplification of these syndromes [7]. Several studies have reported that an excessive inflammatory response and increased inflammatory markers predict adverse outcomes in pneumonia secondary to sepsis, lung injury and ARDS [8]–[11]. There are supportive data to suggest that 30-day mortality in patients with pneumonia is directly caused by pneumonia rather than due to co morbid conditions [3]–[4]. Statins have pleiotropic effects – immunomodulatory [12], anti-inflammatory, anti-thrombotic [13] and a direct microbicidal action [14]; all of which may have potential beneficial role in the prevention and treatment of CAP.

Patients with pneumonia also are at increased risk for cardiac events secondary to increased inflammatory cytokines which could lead to increased thrombosis [15]–[16], interfere with endothelial and ventricular function [17]–[18], cause instability of plaques [19] and promote reperfusion injury [20]. Observational studies have reported increased cardiovascular outcomes in patients with pneumonia [21]–[25]. The proposed beneficial effect of statins in diminishing the risk of cardiac events is due to their anti-inflammatory effect, rather than due to their lipid-lowering properties [26]. Recently, three meta-analyses have shown beneficial roles of statins in the prevention and treatment of several different types of infections [27]–[29].

There have been a number of observational studies evaluating the role of statins in the prevention and treatment of pneumonia [30]–[47]. A recently published meta-analysis did not find an association between the use of statins and prevention of pneumonia when using unadjusted data; but did find an association using adjusted data. This reveals the significance of potential confounders in this association [48]. Another recent meta-analysis suggested a beneficial role of statins in the management of pneumonia [49]. Nevertheless, this review had important limitations, namely ignoring the significant between-study heterogeneity, and publication bias and thus, overestimating the true association.

Another recent meta-analysis of randomized controlled trials (RCT) has also suggested unmeasured confounding as one of the reasons for the apparent beneficial effect of statins in the context of infections [50]. Currently, there are no RCT addressing the role of statins in CAP (www.clinicaltrials.gov).

Given this ongoing controversy, we performed a contemporary systematic review and meta-analysis that addressed the role of statins in the prevention or treatment of CAP and used novel methodology. We added unique approaches to adjust for publication bias as well as explored the potential effect of unknown confounders. We used the Grades of Recommendation, Assessment, Development and Evaluation (GRADE) framework to interpret our findings [51].

## Methods

### Data Sources and Search Strategy

The systematic review was carried out in accordance to the meta-analysis of observational studies in epidemiology and preferred reporting items for systematic reviews and meta-analyses guidelines [52]–[53].

The search strategies were developed in Ovid MEDLINE, and translated to match the subject headings and keywords for Ovid EMBASE, Cochrane database, ISI Web of Science, and Scopus from database inception through December 5, 2011. The subject heading hydroxyl-methyl-glutaryl-CoA reductase inhibitors, including more specific statin subject headings, and keywords for the specific statins: hmgadjcoa or atorvastatin or cerivastatin or compactin or dalvastatin or fluindostation or lovastatin or mevinolin* or monacolin* or pitavastatin or pravastatin or rosuvastatin or simvastatin were matched to subject headings for all respiratory tract infections, pneumonia, CAP, lower respiratory tract infections and chronic obstructive pulmonary disease. There was no restriction of language. All results were downloaded into EndNote 7.0 (Thompson ISI ResearchSoft, Philadelphia), a bibliographic database manager, and duplicate citations were identified and removed. Two authors (A.R.K & A.B.A) independently assessed the eligibility of identified studies.

### Study Selection

The results that were further evaluated were limited to clinical trials, observational studies, case series, and any study that focused on association of statin use and prevention, or prophylaxis, or outcome of CAP and reported an adjusted effect-estimate for this association. Published abstracts or unpublished data was not included as it has been reported that there is discrepancy between published and unpublished data [54]–[55].

We did not specify a priori CAP definition or statins use and they were accepted as defined in individual studies. The outcome was either the incidence of CAP or all cause mortality within the stipulated period of time after an index episode of CAP.

### Data Extraction

Two reviewers (A.R.K. and A.B.A.) independently extracted data on a predefined data collection form. Disagreements between reviewers that could not be resolved by consensus were resolved by a third reviewer (I.M.T).

Extracted data included the following: geographical population under study, subject characteristics, statins intake definition and ascertainment, outcome definitions for prevention and treatment studies and adjusted effect-estimates based on analytical model used in each study. In studies which had multiple adjustments for effect-estimate, the final composite adjusted effect-estimate was used. Since we have focused our analysis on adjusted estimates, we did not attempt to get subgroup data from authors as these data are biased by confounding.

### Quality Assessment

Two reviewers (M.A.G. and A.R.K) independently assessed the methodological quality of selected studies using the Newcastle-Ottawa Quality Assessment Scale for cohort and case-control studies. This scale is used to explore selection bias and comparability between the exposed and unexposed groups, outcome assessment, and attrition bias [56]. Disagreements between reviewers that could not be resolved by consensus were resolved by a third reviewer (I.M.T).

We used the GRADE framework to interpret our findings. The Cochrane Collaboration has adopted the principles of the GRADE system for evaluating the quality of evidence for outcomes reported in systematic reviews [51]. For purposes of systematic reviews, the GRADE approach defines the quality of a body of evidence as the extent to which one can be confident that an estimate of effect or association is close to the quantity of specific interest. Quality of a body of evidence involves consideration of within-study risk of bias (methodological quality), directness of evidence, heterogeneity, precision of effect-estimates and risk of publication bias [51].

### Data Synthesis and Statistical Analysis

The effect-estimates of prevention and treatment studies were pooled separately using the DerSimonian-Laird random-effects model [57] with corresponding Forest plots.

Cochran’s Q test was used to assess heterogeneity among studies, and was complemented by the *I* ^2^ statistic [58]. The influence of a range of study-level and aggregated individual-level parameters on the observed statin effect was investigated by means of meta-regression. Seven potential confounders were considered; five categorical that included geographical population under study (North America vs. European), setting (general practices vs. others), study design (case control vs. cohort), age of the patient (<65 vs. all others), industry sponsored (yes/no or undisclosed) and two continuous variable that included the impact factor of the journal in which the study was published and log of the standard error of the effect-estimate.

### Publication Bias

Contour-enhanced funnel plots [59] were constructed and Egger’s precision test (weighted linear regression) [60] was done to assess funnel plot asymmetry and publication bias. A novel method of regression adjustment of publication bias was used [61]. This model consistently outperforms the conventional ‘trim and fill’ method. For the comparison, the Trim and Fill [62] adjusted effect-estimate was added to the enhanced contour funnel plot.

### Residual Confounding

The possible influence of unknown confounders (residual confounding) was investigated by a novel rule-out approach [63]. This approach stipulates the influence of a hypothetical confounder and determines what characteristics this confounder must have to fully account for the observed association between statin use and the outcome of interest. The hypothetical confounder is characterized by its association to statin use (OR_EC_, odds ratio of exposure to the confounder) and its association to the outcome (RR_CO_, relative risk of outcome in individuals exposed to the confounder). For this analysis, the absolute risk in the pooled, non-exposed group was used for conversion of odds ratio to relative risk using the method described by Zhang and Yu [64]. Separate analyses were performed to demonstrate what levels of OR_EC_ and RR_CO_ would be required to fully explain the observed association between statins and outcome for different hypothetical prevalence of the unknown confounder (PC = 0.2, PC = 0.5) before and after adjustment for publication bias.

All analyses were conducted using Stata version 12 statistical software (StataCorp, Texas).

## Results

### Identification of Studies

**Figure 1:**
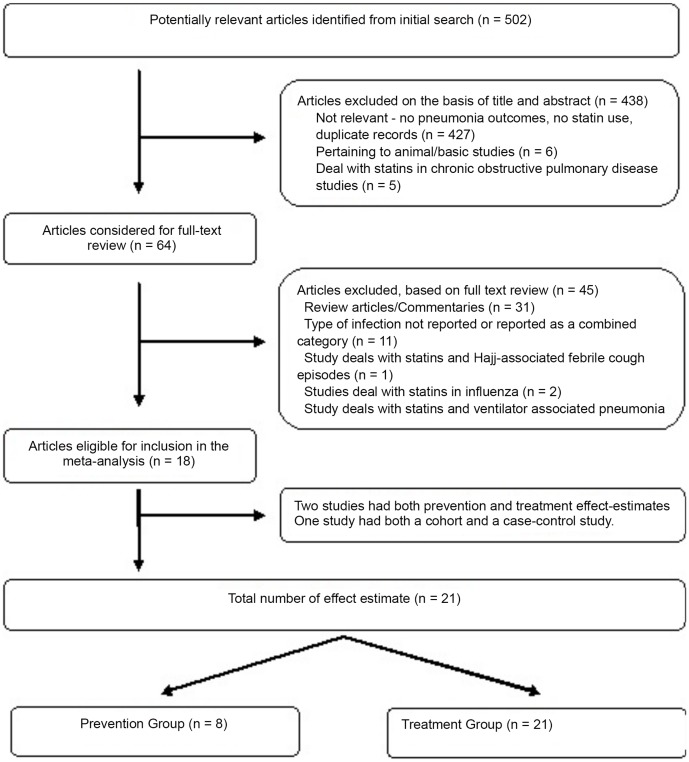
Flow diagram of eligible studies.

The literature search identified 502 publications, out of which 18 were eligible for inclusion in the analysis [30]–[47] (Figure 1). Two studies had both prevention and treatment arms and were included in both the prevention and the treatment groups [36]–[37] and one article comprised of a cohort study and a case-control study [38] and was considered as two studies. One study [40] did not distinguish the cause of death, but was included in the analysis as previous studies have reported that the 30-day mortality is primarily due to CAP rather than other causes [3]–[4]. The only trial on simvastatin in CAP has been suspended for unspecified reasons [65].

Therefore, a total of 18 studies with 21 effect-estimates were included in the final analysis. These studies were divided into two groups; eight studies addressed the role of statins in development of pneumonia [30]–[37]; while thirteen studies addressed the role of statins in outcome of pneumonia [36]–[47] (Supporting Information - Table S1: prevention and treatment groups).

Quality assessment of all included cohort and case-control studies revealed that the studies were of good methodological quality (Supporting Information - Tables S6–S7).

There was excellent agreement for the inclusion of the studies, data abstraction and quality assessment between the reviewers (kappa statistic being 1.0, 1.0 and 0.91 respectively).

### Prevention Group

#### Study characteristics

Supporting Information - Table S1 summarizes the characteristics of the 8 (5 case-control; 3 cohort) studies included in the prevention group [30]–[37]. The studies were conducted in the United Kingdom [31]–[36], United States [30] and Canada [37]. Seven were multi-center [31]–[37]. All of the included studies were population-based and the majority included general practice databases. The relevant outcome of interest in all studies was the development of pneumonia (Supporting Information - Table S2). The effect-estimates of included studies had been adjusted for various confounders (Supporting Information - Table S3).

#### Meta-analysis

**Figure 2:**
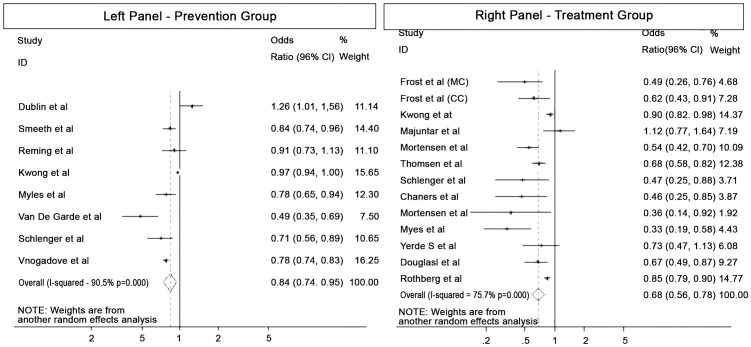
Forest plot of prevention group (left panel) and treatment group (right panel) in the meta-analysis with random effects model. Estimated odds ratios (OR) for the association of (statin use vs. non-use) and development of Pneumonia; CI- indicates Confidence Interval.

A random-effects model was used for the meta-analysis due to substantial between-study heterogeneity (Cochran Q test, p<0.000; I^2^ = 90.5%). It resulted in a pooled effect-estimate of 0.84 (95% CI, 0.74–0.95), suggesting a protective effect of statins against the development of CAP (Figure 2, left panel).

There was visual asymmetry in the contour funnel plot but the number of studies was small, (Supporting Information - Figure S1), and the Egger’s test did not show evidence of publication bias (t = −1.65, p = 0.160).

The univariate meta-regression analyses showed that none of the considered variables were significantly related to the effect-estimate at 5% level of significance.

#### Adjustment for residual confounding

**Figure 3:**
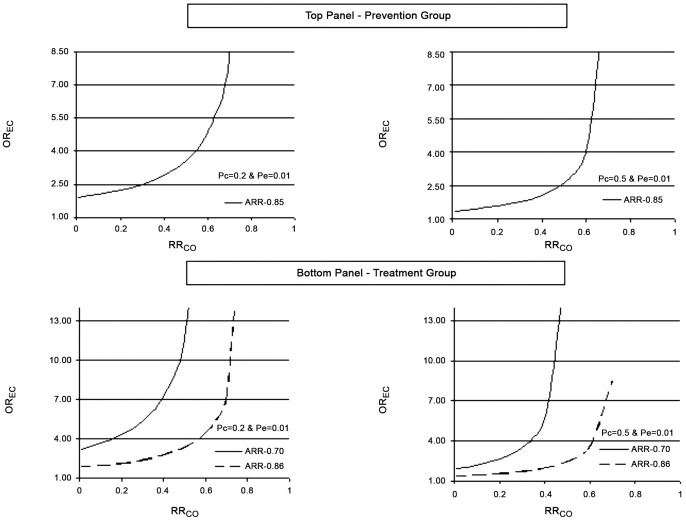
Influence of a hypothetical confounder present in 20% (Left side, both panels) and 50% (Right side, both panels) of the study population, unaccounted for in the adjustments already performed in the individual studies (Prevention group – top panel; Treatment group – bottom panel). The graphs indicate what combinations of OREC and RRCD that would be necessary for the confounder to fully account for the observed association between, (1) Statin use and development of pneumonia (ARR = 0.85)in the prevention group and; (2) Statin use and mortality associated with pneumonia before (ARR = 0.70; Solid Line) and after (ARR = 0.86; Dashed Line) adjustment for publication bias) in the treatment group. *Abbreviations: OREC, odds ratio of exposure to the confounder in statin non-users vs. statin users; RRCD, relative risk of development of pneumonia in individuals exposed to the confounder vs. non-exposed.*

Sensitivity analysis to explain the potential effect of residual confounding was evaluated using the apparent relative risk (ARR = 0.85) of statin users vs. non-users to prevent CAP. At the prevalence of (PC = 0.20), even a very strong confounder causing a 99.9% decrease in CAP risk would have to be severely imbalanced between statin users vs. non-users (OR_EC_ = 1.90) to fully account for the observed RR of 0.85 (Figure 3, left top panel). The top right panel for the figure 3 illustrates the same relationship for a very common confounder (PC = 0.50).

#### Number Needed to Treat (NNT)

The number needed to treat (NNT) was estimated by using the pooled OR from the meta-analysis and based on the incidence of CAP ranging from 3–40/1000 population [1]. The NNT would be 2089 (95% CI; 1285–6686) and 162 (95% CI; 100–520) for an incidence of CAP to 40 per 1000/year, respectively [3].

### Treatment Group

#### Study characteristics

Supporting Information - Table S1 summarizes the characteristics of the 13 studies (one case-control; 12 twelve cohort) included in the treatment group [36]–[47]. The studies were conducted in the United States [38]–[40], [46]–[47] United Kingdom [36], [43]–[45], Canada [37], [42] and Denmark [41]. Eleven of them were multi-center [36]–[38], [40]–[42], [44]–[47]. Statin use was ascertained by review of computerized medical records or pharmacy databases. The relevant outcome of interest was mortality after the diagnosis of pneumonia (either in-hospital or within a stipulated period of time). Most of the studies reported either in-hospital or 30 day mortality except Yende et al [46] who reported a 90 day mortality and Douglas and colleagues who reported a 6 month mortality [45] (Supporting Information - Table S4).

The effect-estimates of the included studies had been adjusted for various confounders (Supporting Information - Table S5).

#### Meta-analysis

A random-effects model yielded a pooled effect-estimate of 0.68 (95% CI, 0.59–0.78), suggesting that statin use was significantly associated with reduced mortality in patients with CAP. There was substantial between-study heterogeneity (Cochran Q test, p<0.000 and I^2^ = 75.7%) (Figure 2, right panel).

The univariate meta-regression analyses showed that the variable “standard error of the effect-estimate” among the considered variables for meta-regression was significantly related to the effect-estimate at 5% level of significance. This suggests a stronger association between statin use and risk of CAP in studies with large standard errors.

**Figure 4:**
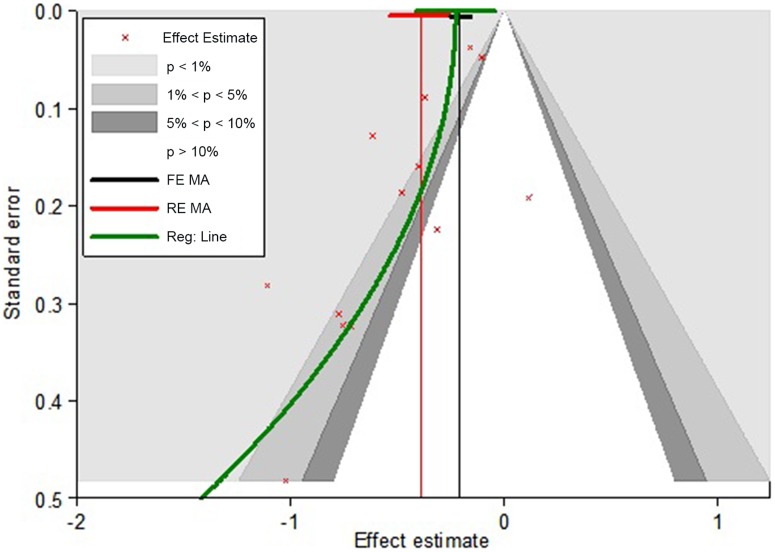
Contour enhanced funnel plot with regression adjustment model for weighted average of the effect -estimate (treatment cohort). Contour enhanced funnel plots with implementation of regression adjustment model (green line); The contour lines differentiate the significance and non-significance regions in the plot at 1%, 5% and 10% significance levels.

Figure 4 displays a contour-enhanced funnel plot with the corresponding fixed effect (FE) and random effect (RE) meta-analyses pooled estimates providing a weighted average of effect size across studies of 0.81 (95% CI, 0.78–0.85) and 0.68 (0.59–0.78), respectively. There was visual evidence of funnel asymmetry and Egger’s test confirms the presence of publication bias, P = 0.014. Hence, a novel regression based method was used to adjust for publication bias (Figure 4). This produced an adjusted average effect-estimate of 0.80 (95% CI, 0.66–0.96).

#### Adjustment for residual confounding

Sensitivity analysis to explore the potential effect of residual confounding was evaluated using the apparent relative risk of statin users vs. non-users, before (ARR = 0.7) and after adjustment for publication bias (ARR = 0.80). At the prevalence of (PC = 0.20) even a very strong confounder causing a 99.9% decrease in mortality would have to be highly imbalanced between statin users vs. non-users (OR_EC_ = 3.21) to fully account for the observed RR of 0.70 (Figure 3, bottom left panel). Similarly Figure 3, bottom right Panel illustrates the relationship for a very common confounder of (PC = 0.50).

#### Number Needed to Treat (NNT)

The NNT estimated by using the pooled OR from the meta-analysis and based on the mortality of CAP being around 10% at 30 days would be 73, (95% CI: 48–158) [1].

## Discussion

In this rigorously conducted systemic review and meta-analysis, we observed that statins use is associated with a beneficial effect in both the prevention and treatment of CAP. This association constitutes very low quality evidence as per the GRADE framework [51]. Factors that negatively influence the quality of the evidence include the observational design, presence of significant heterogeneity, and evidence of publication bias.

Although the effect estimates imply that statins lower the odds of both developing CAP and death related to CAP, we found that the effect estimates were influenced by other sources of bias besides the ones adjusted for in the individual studies. We were able to identify publication bias in the treatment group as opposed to Chopra and colleagues [49] and adjusted for it by a novel regression method. We found that around half of the apparent beneficial effect of statins could be attributed to publication bias.

Our analysis is in agreement with some recently published systematic reviews, but does have some important differences [48]–[49]. First, our meta-analysis did not find any publication bias in the prevention group to evaluate the role of statins in the development of pneumonia. Second, in contrast to the study by Chopra et al [49] we used meta-regression to explore sources of heterogeneity, examined the effect of publication bias using contour-enhanced funnel plot [59] and used a novel regression-based method to adjust the pooled estimate for publication bias. Third, we examined the potential effect of a residual confounding on the observed association using the rule-out approach. We used the GRADE framework to interpret the findings and draw conclusions [51]. The Cochrane Collaboration has adopted the principles of the GRADE system for evaluating the quality of evidence for outcomes reported in systematic reviews [51].

The association between statins use and infection risk and outcomes continues to be controversial. While several systematic reviews of observational studies support this association [27], [29], a recent meta-analysis of RCTs [50] failed to prove a beneficial role of statins in the prevention of infection in a pooled analysis of eligible trials. This study was limited by the lack of reporting of infection-related events in the majority of statin trials. In addition, the majority of included trials did not describe the type of infection in the cohort [50]. On the other hand, a recent open-label controlled trial showed pravastatin to have a favorable outcome on the frequency of development of ventilator associated pneumonia (VAP) and associated overall mortality in statin-naïve patients in the ICU on mechanical ventilation [66]. These beneficial effects were found in the sub-group of patients who were more critically ill.

Our meta-analysis has several strengths. It was focused on CAP and thus reduced the variability in the study populations with panoply of infections. We also supplemented our analysis with novel approaches to adjust for publication bias and unmeasured confounders. We tried to quantify the effects of other sources of bias in order to secure robust conclusions. All included studies were population-based and the majority were multi-center studies; thus, the results have applicability to the general population. We also calculated the NNT so as to measure the effectiveness of statins in the general population.

There are limitations to our work. First, observational studies are subject to inherent limitations in the study design leading to unmeasured differences in the study population and unmeasured confounders despite all possible adjustments. We have used a novel approach to address the concern of unmeasured confounding. This approach has been previously applied to address the issue of statins and mortality in bacterial infections [29]. Second, use of administrative records for classification of pneumonia could lead to case ascertainment bias of CAP [33]. Third, the use of statins was based on electronic and prescription records, rather than by actual use by the patient. Fourth, the results may have been affected by the “healthy user effect” [41], [44], [67] or even selective underuse of statins in high risk groups [30]. However, we tried to adjust for any unmeasured confounders affecting the pooled effect estimate. Fifth, there is presence of publication bias in the treatment group; we have tried to use a novel regression method to adjust the pooled effect-estimate secondary to publication bias. Lastly, there was substantial amount of heterogeneity in the included studies. We used a random-effects model to minimize heterogeneity. Some likely sources of heterogeneity were investigated by means of a meta-regression, but were not to be found significant. There are many patient level parameters which may have led to substantial heterogeneity – time to antibiotic delivery, place of care i.e. floor versus the ICU, frailty, nursing home status and time duration from the index episode of pneumonia to mortality. Investigating these variables is only possible with individual patient data meta-analysis. Moreover, the heterogeneity would mainly have an effect on the magnitude of the pooled estimate rather than its direction (the effect-estimates of 7 out of 8 prevention studies and 12 out of 13 treatment studies support a protective association).

Given the potential benefit of statins, and relatively low speculated NNT, a dedicated RCT is warranted to further examine their role in CAP prevention and treatment. To be feasible, future RCTs have to focus on high risk groups for CAP or mortality from CAP such as the elderly, the immunocompromised host or patients with significant co-morbid status. Performing an RCT for this patient population will require a relatively small sample size of patients to be enrolled as opposed to mega large trials needed to study statins role in the general population. Presently, because of the low quality of evidence available in the favor of use of statins, we do not recommend initiating statins in patients admitted with suspected CAP. We do suggest, however, continuing pre-admission statins in patients admitted with pneumonia.

### Conclusions

Our meta-analysis reveals an association between statins and the risk and mortality of CAP. However, the results constitute very low quality evidence as per the GRADE framework due to observational study design, heterogeneity and publication bias. Given the biological plausibility of our findings and the high burden and mortality of CAP, randomized, placebo-controlled trials are warranted to further define the utility of statins in CAP, especially in select high risk groups.

## Supporting Information

Figure S1 **Contour enhance funnel plot of the association between the effect estimates and its standard errors (prevention group).** The contour lines differentiate the significance and non-significance regions in the plot at 1%, 5% and 10% significance levels.
(DOC)

Table S1 **Characteristics of the included Prevention and Treatment Groups.**
(DOC)

Table S2 **Analytical Approach and Results of included studies in the Prevention Group.**
(DOC)

Table S3 **Confounders Adjusted for in Statin Prevention Group.**
(DOC)

Table S4 **Analytical Approach and Results of included studies in the Treatment Group.**
(DOC)

Table S5 **Confounders Adjusted for in Statin Treatment Group.**
(DOC)

Table S6 **Modified Newcastle-Ottawa Quality Assessment Scale for Cohort Studies included in the Meta-analysis.** The criteria used for selection, comparability and outcome were - *Selection was based on representativeness of the exposed cohort, selection of the non-exposed cohort, and ascertainment of exposure; Comparability of cohorts was on the basis of the design or analysis; Outcome assessment was based on the follow up and its adequacy.*
(DOC)

Table S7 **Modified Newcastle-Ottawa Quality Assessment Scale for Case-Control Studies included in the Meta-analysis.** The criteria used for selection, comparability and outcome were - *Selection was based on case definition, representativeness of the cases, selection and definition of controls; Comparability of cases and controls was on the basis of the design or analysis; Outcome was based on ascertainment of exposure of both cases and controls.*
(DOC)

Checklist S1 **PRISMA Checklist.**
(DOC)

## References

[1] Torres A, Rello J (2010) Update in Community-acquired and Nosocomial Pneumonia 2009. AJRCCM. 181: 782–787. [PMID: 20382801]. 10.1164/rccm.201001-0030UP 20382801

[2] Chiou CC, Yu LV (2006) Severe pneumococcal pneumonia: new strategies for management. Curr Opin Crit Care. 12: 470–6. [PMID:16943728]. 10.1097/01.ccx.0000244129.69742.d9 16943728

[3] Mortensen EM, Coley CM, Singer DE, Marrie TJ, Obrosky DS, et al.. (2002) Causes of death for patients with community-acquired pneumonia: results from the Pneumonia Patient Outcomes Research Team cohort study. Arch Intern Med. 162: 1059–1064. [PMID: 11996618]. 10.1001/archinte.162.9.1059 11996618

[4] Mortensen EM, Kapoor WN, Chang CC, et al.. (2003) Assessment of mortality after long-term follow-up of patients with community-acquired pneumonia. Clin Infect Dis. 37: 1617–1624. [PMID: 14689342]. 10.1086/379712 14689342

[5] Waterer GW, Kessler LA, Wunderink RG (2004) Medium-term survival after hospitalization with community-acquired pneumonia. Am J Respir Crit Care Med. 169: 910–914. 10.1164/rccm.200310-1448OC 14693672

[6] Kung HC, Hoyert DL, Xu J, Murphy SL (2008) Deaths: final data for 2005. Natl Vital Stat Rep. 56(10): 1–124. 18512336

[7] Wang H, Ma S (2008) The cytokine storm and factors determining the sequence and severity of organ dysfunction in multiple organ dysfunction syndrome. American Journal of Emergency Medicine. 26: 711–715. [PMID:18606328]. 10.1016/j.ajem.2007.10.031 18606328

[8] Kellum JA, Kong L, Fink MP, Weissfeld LA, Yealy DM, et al.. (2007) Understanding the inflammatory cytokine response in pneumonia and sepsis: results of the Genetic and Inflammatory Markers of Sepsis (GenIMS) study. Arch Intern Med. 167(15): 1655–1663. [PMID: 17698689]. 10.1001/archinte.167.15.1655 PMC4495652 17698689

[9] Menéndez R, Cavalcanti M, Reyes S, Mensa J, Martinez R, et al.. (2008) Markers of treatment failure in hospitalised community acquired pneumonia. Thorax. 63(5): 447–52. [PMID: 18245147]. 10.1136/thx.2007.086785 18245147

[10] Fernandez-Serrano S, Dorca J, Coromines M, Carratalà J, Gudiol F, et al.. (2003) Molecular inflammatory responses measured in blood of patients with severe community-acquired pneumonia. Clin Diagn Lab Immunol. 10: 813–820 [PIMD: 12965910]. 10.1128/CDLI.10.5.813-820.2003 PMC193876 12965910

[11] Antunes G, Evans SA, Lordan JL, Frew AJ (2002) Systemic cytokine levels in community-acquired pneumonia and their association with disease severity. Eur Respir J. 20: 990–995. [PMID: 12412694]. 10.1183/09031936.02.00295102 12412694

[12] Kwak B, Mulhaupt F, Myit S, Mach F (2000) Statins as a newly recognized type of immunomodulator. Nat Med. 6(12): 1399–1402. [PMID:11100127]. 10.1038/82219 11100127

[13] Steiner S, Speid WS, Pleiner J, Seidinger D, Zorn G, et al.. (2005) Simvastatin blunts endotoxin-induced tissue factor in vivo. Circulation. 111(14): 1841–1846. [PMID:15824212]. 10.1161/01.CIR.0000158665.27783.0C 15824212

[14] Catron DM, Lange Y, Borensztajn J, Sylvester MD, Jones BD, et al.. (2004) Salmonella enterica serovar Typhimurium requires nonsterol precursors of the cholesterol biosynthetic pathway for intracellular proliferation. Infect Immun. 72(2): 1036–1042. [PMID:14742551]. 10.1128/IAI.72.2.1036-1042.2004 PMC321618 14742551

[15] Danesh J, Collins R, Appleby P, Peto R (1998) Association of fibrinogen, C-reactive protein, albumin, or leukocyte count with coronary heart disease: meta-analyses of prospective studies. JAMA. 279: 1477–82. [PMID:9600484]. 10.1001/jama.279.18.1477 9600484

[16] Mendall MA, Patel P, Asante M, Ballam L, Morris J, et al.. (1997) Relation of serum cytokine concentrations to cardiovascular risk factors and coronary heart disease. Heart. 78(3): 273–7. [PMID:9391290]. 10.1136/hrt.78.3.273 PMC484930 9391290

[17] Kumar A, Thota V, Dee L, Olson J, Uretz E, et al.. (1996) Tumor necrosis factor alpha and interleukin 1beta are responsible for in vitro myocardial cell depression induced by human septic shock serum. J Exp Med. 183: 949–58. [PMID: 8642298]. 10.1084/jem.183.3.949 PMC2192364 8642298

[18] Mann DL (2002) Inflammatory mediators and the failing heart: past, present, and the foreseeable future. Circ Res. 91: 988–98. [PMID: 12456484]. 10.1161/01.res.0000043825.01705.1b 12456484

[19] Ramirez J, Aliberti S, Mirsaeidi M, Peyrani P, Filardo G, et al.. (2008) Acute myocardial infarction in hospitalized patients with community-acquired pneumonia. Clin Infect Dis. 47(2): 182–187. [PMID: 18533841]. 10.1086/589246 18533841

[20] Shin WS, Szuba A, Rockson SG (2002) The role of chemokines in human cardiovascular pathology: enhance bioloical insights. Atherosclerosis. 160(1): 91–102. [PMID: 11755926]. 10.1016/s0021-9150(01)00571-8 11755926

[21] Corrales-Medina VF, Madjid M, Musher DM (2010) Role of acute infection in triggering acute coronary syndromes. Lancet Infect Dis. 10: 83–92. 10.1016/S1473-3099(09)70331-7 20113977

[22] Musher DM, Rueda AM, Kaka AS, Mapara SM (2007) The association between pneumococcal pneumonia and acute cardiac events. Clin Infect Dis. 45(2): 158–65. [PMID: 17578773]. 10.1086/518849 17578773

[23] Perry TW, Pugh MJ, Waterer GW, Nakashima B, Orihuela CJ, et al.. (2011) Incidence of cardiovascular events after hospital admission for pneumonia. Am J Med. 124(3): 244–51. [PMID: 21396508]. 10.1016/j.amjmed.2010.11.014 PMC3061467 21396508

[24] Corrales-Medina VF, Serpa J, Rueda AM, Giordano TP, Bozkurt B, et al.. (2009) Acute bacterial pneumonia is associated with the occurrence of acute coronary syndromes. Medicine (Baltimore). 88(3): 154–159 [PMID:19440118]. 10.1097/MD.0b013e3181a692f0 19440118

[25] Yende S, D’Angelo G, Mayr F, Kellum JA, Weissfeld S, et al.. (2011) Elevated hemostasis markers after pneumonia increases one-year risk of all cause and cardiovascular deaths. PloS ONE. 6(8): e22847. [PMID:21853050]. 10.1371/journal.pone.0022847 PMC3154260 21853050

[26] Serhan CN, Chiang N, Van Dyke TE (2008) Resolving inflammation: dual anti-inflammatory and pro-resolution lipid mediators. Nat Rev Immunol 8: 349–361. [PMID: 18437155]. 10.1038/nri2294 PMC2744593 18437155

[27] Tleyjeh IM, Kashour T, Hakim FA, Zimmerman VA, Erwin PJ, et al.. (2009) Statins for the Prevention and Treatment of Infections. A Systematic Review and Meta-analysis. Arch Intern Med. 169(18): 1658–1667. [PMID:19822822]. 10.1001/archinternmed.2009.286 19822822

[28] Janda S, Young A, FitzGerald JM, Etminan M, Swiston J (2010) The effect of statins on mortality from severe infections and sepsis: A systematic review and meta-analysis. J Crit Care. 25(4): 656.e7–22. [PMID: 20413251]. 10.1016/j.jcrc.2010.02.013 20413251

[29] Bjorkhem-Bergman L, Bergman P, Andersson J, Lindh JD (2010) Statin Treatment and Mortality in Bacterial Infections – A Systematic Review and Meta-Analysis. PLoS ONE. 19;5(5):e1070. [PMID: 20502712]. 10.1371/journal.pone.0010702 PMC2873291 20502712

[30] Dublin S, Jackson ML, Nelson JC, Weiss NS, Larson EB, et al.. (2009) Jackson LA. Statin use and risk of community acquired pneumonia in older people: population based case-control study. BMJ. 338: b2137. [PMID: 19531550]. 10.1136/bmj.b2137 PMC2697311 19531550

[31] Smeeth L, Douglas I, Hall AJ, Hubbard R, Evans S (2009) Effect of statins on a wide range of health outcomes: a cohort study validated by comparison with randomized trials. Br J Clin Pharmacol. 67(1): 99–109. [PMID: 19006546]. 10.1111/j.1365-2125.2008.03308.x PMC2668090 19006546

[32] Fleming DM, Verlander NQ, Elliot AJ, Zhao H, Gelb D, et al.. (2010) An assessment of the effect of statin use on the incidence of acute respiratory infections in England during winters 1998–1999 to 2005–2006. Epidemiol Infect. 138: 1281–1288. [PMID: 20109259]. 10.1017/S0950268810000105 20109259

[33] Myles PR, Hubbard RB, McKeever TM, Z Pogson, Smith CJP, et al (2009) Risk of community-acquired pneumonia and the use of statins, ace inhibitors and gastric acid suppressants: a population-based case–control study. Pharmacoepidemiology and drug safety. 18: 269–275. 10.1002/pds.1715 19235776

[34] Van de Garde EMW, Hak E, Souverein PC, Hoes AW, van den Bosch JMM, et al.. (2006) Statin treatment and reduced risk of pneumonia in patients with diabetes. Thorax. 61: 957–961. [PMID: 16809409]. 10.1136/thx.2006.062885 PMC2121156 16809409

[35] Vinogradova Y, Coupland C, Hippisley-Cox J (2011) Risk of pneumonia in patients taking statins: population-based nested case-control study. Br J Gen Pr. 61(592): e742–8 [PMID: 220543381]. 10.3399/bjgp11X606654 PMC3207092 22054338

[36] Schlienger RG, Fedson DS, Jick SS, Jick H, Meier CR (2007) Statins and the risk of pneumonia: a population-based, nested case-control study. Pharmacotherapy. 27: 325–32. [PMID: 17316144]. 10.1592/phco.27.3.325 17316144

[37] Kwong JC, Li P, Redelmeier DA (2009) Influenza Morbidity and Mortality in Elderly Patients Receiving Statins: A Cohort Study. PLoS ONE. 4(11): e8087. [PMID: 19956645]. 10.1371/journal.pone.0008087 PMC2778952 19956645

[38] Frost FJ, Petersen H, Tollestrup K, Skipper B (2007) Influenza and COPD Mortality Protection as Pleiotropic, Dose-Dependent Effects of Statins. Chest. 131: 1006–1012. [PMID: 17426203]. 10.1378/chest.06-1997 17426203

[39] Mortensen EM, Pugh MJ, Copeland LA, Restrepo MI, Cornell JE, et al. 92008) Impact of statins and angiotensin converting enzyme inhibitors on mortality of subjects hospitalised with pneumonia. Eur Respir J. 31: 611–617. [PMID: 17959631]. 10.1183/09031936.00162006 17959631

[40] Mortensen EM, Restrepo MI, Anzueto A, Pugh J (2005) The effect of prior statin use on 30-day mortality for patients hospitalized with community-acquired pneumonia. Respiratory Research. 6: 82 [PMID: 16042797]. 10.1186/1465-9921-6-82 PMC1199623 16042797

[41] Thomsen RW, Riis A, Kornum JB, Christensen S, Johnsen SP, et al.. (2008) Preadmission Use of Statins and Outcomes After Hospitalization With Pneumonia. Arch Intern Med. 168(19): 2081–2087. [PMID: PMID:18955636]. 10.1001/archinte.168.19.2081 18955636

[42] Majumdar SR, McAlister FA, Eurich DT, Padwal RS, Marrie TJ (2006) Statins and outcomes in patients admitted to hospital with community acquired pneumonia: population based prospective cohort study. BMJ. 11; 333(7576): 999. [PMID: 17060337]. 10.1136/bmj.38992.565972.7C PMC1635620 17060337

[43] Chalmers JD, Singanayagam A, Murray MP, Hill AT (2008) Prior statin use is associated with improved outcomes in community-acquired pneumonia. Am J Med. 121: 1002–1007. [PMID:18954848]. 10.1016/j.amjmed.2008.06.030 18954848

[44] Myles PR, Hubbard RB, Gibson JE, Pogson Z. Smith CJP, et al.. (2009) The impact of statins, ACE inhibitors and gastric acid suppressants on pneumonia mortality in a UK general practice population cohort. Pharmacoepidemiology and drug safety. 18: 697–703. [PMID:19455553]. 10.1002/pds.1769 19455553

[45] Douglas I, Evans S, Smeeth L (2011) Effect of statin treatment on short term mortality after pneumonia episode: cohort study. BMJ. 342: d1642. [PMID: 21471172]. 10.1136/bmj.d1642 PMC3071610 21471172

[46] Yende S, Milbrandt EB, Kellum JA, Kong L, Delude RL, et al.. (2011) Understanding the potential role of statins in pneumonia and sepsis. Crit Care Med. 39(8): 1871–8. [PMID: 21516038]. 10.1097/CCM.0b013e31821b8290 PMC3139804 21516038

[47] Rothberg MB, Bigelow C, Pekow PS, Lindenauer PK (2011) Association Between Statins Given in Hospital and Mortality in Pneumonia Patients. J Gen Intern Med. [Epub ahead of print] [PMID: 21842322]. 10.1007/s11606-011-1826-2 PMC3286569 21842322

[48] Kwok CS, Yeong JK, Turner RM, Cavallazzi R, Singh S, et al.. (2012) Statins and associated risk of pneumonia: a systematic review and meta-analysis of observational studies. Eur J Clin Pharmacol. 68(5): 747–55. Epub 2011 Nov 15. 10.1007/s00228-011-1159-4 22083167

[49] Chopra V, Rogers MA, Buist M, Govindan S, Lindenauer PK, et al.. (2012)Is Statin Use Associated with Reduced Mortality After Pneumonia? A Systematic Review and Meta-analysis Am J Med. [Epub ahead of print]. 10.1016/j.amjmed.2012.04.011 22835463

[50] Van den Hoek HL, Bos WJ, de Boer A, van de Garde EM (2011) Statins and prevention of infections: systematic review and meta-analysis of data from large randomised placebo controlled trials BMJ. 29; 343: d7281. [PMID: 22127443]. 10.1136/bmj.d7281 PMC3226140 22127443

[51] Schünemann HJ, Oxman AD, Vist GE, Higgins JPT, Deeks JJ, et al. (2008) Chapter 12: Interpreting results and drawing conclusions. In: Higgins JPT, Green S (editors), *Cochrane Handbook for Systematic Reviews of Interventions.* Version 5.0.1 [updated September 2008]. The Cochrane Collaboration. Available: www.cochrane-handbook.org.

[52] Stroup DF, Berlin JA, Morton SC, Olkin I, Williamson GD, et al.. (2000) Meta-analysis of obser vational studies in epidemiology (MOOSE) group. JAMA. 19;283(15): 2008–12. [PMID: 10789670]. 10.1001/jama.283.15.2008 10789670

[53] Liberati A, Altman DG, Tetzlaff J, Mulrow C, Gøtzsche PC, et al.. (2009) The PRISMA statement for reporting systematic reviews and meta-analyses of studies that evaluate healthcare interventions: explanation and elaboration. BMJ. 339: b2700. [PMID: 19622552]. 10.1136/bmj.b2700 PMC2714672 19622552

[54] Taddio A, Pain T, Fassos FF, Boon H, Ilersic AL, et al.. (1994) Quality of nonstructured and structured abstracts of original research articles in the British medical journal, the Canadian medical association journal and the journal of the American medical association. CMAJ. 150(10): 1611–1615 [PMID: 8174031]. PMC1336964 8174031

[55] Scherer RW, Langenberg P and von Elm E (2007) Full publication of results initially presented in abstracts. Cochrane Database of Systematic Reviews. 18; (2): MR000005. [PMID: 17443628]. 10.1002/14651858.MR000005.pub3 17443628

[56] Wells G, Shea B, O’Connell D, J Peterson, V Welch, et al. (2011) The Newcastle- Ottawa scale (NOS) for assessing the quality of nonrandomized studies in meta-analysis. Ottawa, Ontario: The Ottawa Health Research Institute. Available: http://www.ohri.ca/programs/clinicalepidemiology/nosgen.doc. Accessed 2011 Sept 13.

[57] DerSimonian R, Laird N (1986) Meta-analysis in clinical trials. Control Clin Trials. 7(3): 177–188. 10.1016/0197-2456(86)90046-2 3802833

[58] Higgins JP, Thompson SG (2002) Quantifying heterogeneity in a meta-analysis. Stat Med. 21(11): 1539–1558. 10.1002/sim.1186 12111919

[59] Peters JL, Sutton AJ, Jones DR, Abrams KR, Rushton L (2008) Contour-enhanced meta-analysis funnel plots help distinguishing publications bias from other causes of asymmetry. J Clin Epidemiol. 61: 991–996. 10.1016/j.jclinepi.2007.11.010 18538991

[60] Egger M, Davey Smith G, Schneider M, Minder C (1997) Bias in meta-analysis detected by a simple, graphical test. BMJ. 315(7109): 629–634. 10.1136/bmj.315.7109.629 PMC2127453 9310563

[61] Moreno SG, Sutton AJ, Ades AE, Stanley TD, Abrams KR, et al.. (2009) Assessment of regression-based methods to adjust for publication bias through a comprehensive simulation study. BMC Med Res Methodol. 9: 2. [PMID: 19138428]. 10.1186/1471-2288-9-2 PMC2649158 19138428

[62] Duval S, Tweedie R (2000) A Nonparametric “Trim and Fill” Method of Accounting for Publication Bias in Meta-Analysis. Journal of the American Statistical Association. 95(449): 89–98.

[63] Schneeweiss S (2006) Sensitivity analysis and external adjustment for unmeasured confounders in epidemiologic database studies of therapeutics. Pharmacoepidemiol Drug Saf. 15(5): 291–303. [PMID:16447304]. 10.1002/pds.1200 16447304

[64] Zhang J, Yu KF (1998) What’s the relative risk? A method of correcting the odds ratio in cohort studies of common outcomes. JAMA. 18;280(19): 1690–1. [PMID: 9832001]. 10.1001/jama.280.19.1690 9832001

[65] Impact of Statins on Cytokine Expression in Pneumonia. Available: http://clinicaltrials.gov/ct2/show/NCT00946166?term=pneumonia%2Cstatins&rank=1. 2012 Jun 11.

[66] Makris D, Manoulakas E, Komnos A, Papakrivou E, Tzovaras N, et al.. (2011) Effect of pravastatin on the frequency of ventilator-associated pneumonia and on intensive care unit mortality: open label, randomized study. Crit Care Med. 39(11): 2440–6. [PMID 21725239]. 10.1097/CCM.0b013e318225742c 21725239

[67] Glynn RJ, Schneeweiss S, Wang PS, Levin R, Avorn J (2006) Selective prescribing led to overestimation of the benefits of lipid-lowering drugs. J Clin Epidemiol. 59: 819–28. [PMID:16828675]. 10.1016/j.jclinepi.2005.12.012 16828675

