# Supplementary material for: The Role of Statins in Prevention and Treatment of Community Acquired Pneumonia: A Systematic Review and Meta-Analysis
Source: PLoS One. 2013 Jan 7;8(1):e52929. doi: 10.1371/journal.pone.0052929 (PMC3538683; doi:10.1371/journal.pone.0052929)
Supplement: Table S1 — Characteristics of the included Prevention and Treatment Groups. (DOC) [file pone.0052929.s002.doc]

| **Table S1. Characteristics of Included Prevention and Treatment Groups** | | | | | | | | | | | |
| --- | --- | --- | --- | --- | --- | --- | --- | --- | --- | --- | --- |
| **Source, Study Period** | **Country** | | **Centers** | **Setting** | **Condition** | | **Inclusion Criteria** | **Study Design** | **Statin use Ascertainment** | **Follow-up** | |
| **Prevention Group** | | | | | | | | | | | |
| Dublin et al ^A1^  2000-2003 | | US | Single | Integrated healthcare delivery system, | | CAP | Age 65-94 y; No CA, HIV, ESRD, immunosuppression | Population based case-control | Computerized pharmacy database | - | |
| Smeeth et al^A2^  1995-2006 | | UK | Multicenter | 303 General practices, THIN database | | CAP |  | Population based cohort | Computerized medical records | 3.8-4.2 | |
| Fleming et al^A3^  98-99 to'05-06 | | UK | Multicenter | 25/102 Practices in WRS of RCGP | | CAP | Age ≥ 45 y; with CVD | Population based retrospective cohort | Electronic databases; Prescription records | - | |
| Myles et al^A4^  2001-2002 | | UK | Multicenter | 300 General practices, THIN database | | CAP; including HAP, HIV/ PCP | Age > 40 y | Population based matched case control | Prescription records | - | |
| Van De Garde et al ^A5^  1987-2001 | | UK | Multicenter | 600 GPs; General practice research database (GPRD) | | CAP | Age > 18 y | Retrospective, case control | Prescription records | - | |
| Vinogradova et al ^A6^  1996-2005 | | UK | Multicenter | 500 GPs; Qresearch database | | CAP | Age > 45 y | Population based nested case control | Prescription records | - | |
| **Prevention and Treatment Groups** | | | | | | | | | | | |
| Schlienger et al^A7^  1995-2002 | | UK | Multicenter | General practice research database (GPRD) | | CAP | Age > 30 y; No HIV, immunosuppression | Retrospective, nested case control | Computerized prescription database | | 30 day |
| Kwong et al^A8^  1996-2006 | | Canada | Multicenter | Multiple administrative healthcare databases | | CAP | Age > 65 y; received influenza vaccination | Retrospective cohort | Prescription records | | 30 day |
| **Treatment Group** | | | | | | | | | | | |
| Frost et al^A9^  1992-2003 | | US | Multicenter | Moderate-size HMOs, Lovelace database | | CAP; Influenza | Inpatient | Matched cohort study;  Case-control study | Pharmacy dispensing data | | In hospital |
| Mortensen et al^A10^ 1999-2002 | | US | Single | VA outpatient and inpatient administrative data | | CAP; maybe sepsis/resp. failure | Age≥ 65 y; hospitalized with pneumonia; received 1 statin prescription within 90 days | Retrospective cohort | Pharmacy database | | 30 day |
| Mortensen et al ^A11^  1999-2002 | | US | Multicenter | Tertiary care teaching hospitals, Texas | | CAP; maybe sepsis/resp. failure | Age > 18 y; pneumonia | Retrospective cohort | Electronic medical record; Outpatient charts | | 30 day |
| Thomsen et al^A12^  1997-2008 | | Denmark | Multicenter | Hospitals in counties of Aarhus and North Jutland | | CAP | Age > 15 y; discharge diagnosis of pneumonia | Population based cohort | Regional prescription databases | | 90 day |
| Majumdar et al ^A13^  2000-2002 | | Canada | Multicenter | Capital Health integrated health system, Alberta | | CAP | Age > 17 y; inpatient; no TB, CF, immunocompromised | Population based prospective cohort | Medical charts; patient/proxy interviews | | In hospital |
| Chalmers et al ^A14^  2005-2007 | | UK | Single | NHS Lothian university hospitals division, | | CAP | CAP (CXR/ signs/symptoms) No HAP, malignancy, CLD, immunosuppression | Prospective observational study | Self reported prescription; confirmed by contacting GP | | 30 day |
| Myles et al ^A15^  2001-2002 | | UK | Multicenter | 300 General practices, THIN database | | CAP; include TB, HAP, HIV/PCP | Age > 40 y | Population based cohort | Prescription records | | 30 day |
| Douglas I et al ^A16^ 1995-2006 | | UK | Multicenter | 300 General practices, THIN database | | CAP | Age > 40 y, statin prescription at least 60d prior to pneumonia | Population based cohort, Propensity score | Prescription records | | 6 month |
| Yende S et al ^A17^ 2001-2003 | | US | Multicenter | Emergency department of 28 US hospitals | | CAP | Clinical/radiological diagnosis of CAP | Multicenter Inception cohort, prospective | -- | | 90 day |
| Rothberg MB et al ^A18^ 2003-2005 | | US | Multicenter | 376 acute care facilities | | CAP | Age > 18 y; pneumonia with or without sepsis/resp failure | Retrospective cohort | Electronic databases; Prescription records | | In hospital |
