# Supplementary material for: The Role of Statins in Prevention and Treatment of Community Acquired Pneumonia: A Systematic Review and Meta-Analysis
Source: PLoS One. 2013 Jan 7;8(1):e52929. doi: 10.1371/journal.pone.0052929 (PMC3538683; doi:10.1371/journal.pone.0052929)
Supplement: Table S2 — Analytical Approach and Results of included studies in the Prevention Group. (DOC) [file pone.0052929.s003.doc]

| **Table S2. Analytical Approach and Results of included studies in the Prevention Group** | | | | |
| --- | --- | --- | --- | --- |
| **Results :(Adjusted Effect**  **Estimates, 95% CI)** | **Outcome (Risk of**  **developing Pneumonia)** | **Analytical Method** | **Sample Size** | **Source** |
| Adjusted OR, 1.26 (1.01 – 1.56) | Pneumonia | Conditional logistic regression | Statin user cases: 181 of 1125  Statin user controls: 327 of 2235  Case matching: gender, age, calendar year,  duration of pneumonia-free follow-up | Dublin et al ^30^ |
| Adjusted OR, 0.84 (0.74 – 0.95) | Pneumonia | Conditional logistic regression  and propensity score | Statin users: 129288  Statin nonusers: 600241 | Smeeth et al^31^ |
| Adjusted OR, 0.91 (0.73 – 1.13) | Pneumonia | Logistic regression | Statin users: 61259  Statin nonusers: 267622 | Fleming et al^32^ |
| Adjusted OR, 0.78 (0.65 – 0.94) | Pneumonia | Conditional multiple logistic regression | Statin user cases: 178 of 3709  Statin user controls: 1050 of 22174  Case matching: gender, age, practice, | Myles et al^33^ |
| Adjusted OR, 0.49 (0.35 – 0.69) | Pneumonia | Conditional logistic regression | Statin user cases: 50 of 4719  Statin user controls: 318 of 15322  Case matching: gender, age,  general practice, pneumonia dx date | Van De Garde et al ^34^ |
| Adjusted OR 0.78 (0.74-0.83) | Pneumonia | Conditional logistic regression | Pneumonia cases 17755  Controls 80484  Case matching: age, gender | Vinogradova et al ^35^ |
| Adjusted OR, 0.71 (0.56 – 0.89) | Pneumonia -uncomplicated | Conditional logistic regression | Statin user cases: 141 of 1253  Statin user controls: 599 of 4838  Case matching: gender, age,  general practice, pneumonia diagnosis date | Schlienger et al^36^ |
| Adjusted OR, 0.97 (0.94 – 1.00) | Pneumonia | Conditional logistic regression | Statin users: 1120319  Statin nonusers: 1120319 | Kwong et al^37^ |
