# Supplementary material for: The Role of Statins in Prevention and Treatment of Community Acquired Pneumonia: A Systematic Review and Meta-Analysis
Source: PLoS One. 2013 Jan 7;8(1):e52929. doi: 10.1371/journal.pone.0052929 (PMC3538683; doi:10.1371/journal.pone.0052929)
Supplement: Table S3 — Confounders Adjusted for in Statin Prevention Group. (DOC) [file pone.0052929.s004.doc]

| **Table S3. Confounders Adjusted for in Statin Prevention Group** | | | | | |  |
| --- | --- | --- | --- | --- | --- | --- |
| **Source** | **Demographics** | **Co-morbidities ^1^** | **Prior Vaccination** | **Smoking** | **Concurrent Medications^2^** | **Propensity Scores** |
| Dublin et al ^30^ | *+* | *16* | *+* | *+* | *4* | *-* |
| Smeeth et al^31^ | *+* | *12* | *-* | *-* | *11* | *+* |
| Fleming et al^32^ | *+* | *5* | *+* | *+* | *7* | *-* |
| Myles et al^33^ | *+* | *CI* | *-* | *+* | *2* | *-* |
| Van De Garde et al ^34^ | *+* | *4* | *+* | *+* | *3* | *-* |
| Vinogradova et al ^35^ | *+* | *22* | *+* | *+* | *2* | *-* |
| Schlienger et al^36^ | *+* | *9* | *-** | *+* | *2* | *-* |
| Kwong et al^37^ | *+* | *7* | *-* | *-* | *-* | *-* |

*^1^, Number of comorbidities and risk factors defined in the article (Some comorbidities which were not found to be significant in the specific study have not been listed); ^2^, The number of medications for which adjustment was required as they were significant*

*+, The analysis was adjusted for this variable; -, The analysis was not adjusted for this variable*

*CI, Charlson’s Comorbidity index.*

**, Excluded as they were not significant in the univariate analysis.*

*Note – There may have been more than the enumerated comorbidities in the studies but only those for which adjustment performed is included*
