# Supplementary material for: The Role of Statins in Prevention and Treatment of Community Acquired Pneumonia: A Systematic Review and Meta-Analysis
Source: PLoS One. 2013 Jan 7;8(1):e52929. doi: 10.1371/journal.pone.0052929 (PMC3538683; doi:10.1371/journal.pone.0052929)
Supplement: Table S4 — Analytical Approach and Results of included studies in the Treatment Group. (DOC) [file pone.0052929.s005.doc]

| **Table S4. Analytical Approach and Results of included studies in the Treatment Group** | | | | |
| --- | --- | --- | --- | --- |
| **Results :(Adjusted Effect**  **Estimates, 95% CI)** | **Outcome (Pneumonia**  **associated mortality)** | **Analytical Method** | **Sample Size** | **Source** |
| Adjusted OR, 0.47 (0.25 – 0.88) | 30 day mortality | Conditional logistic regression | Statin user cases: 19 of 283  Statin user controls: 131 of 1030  Case matching: gender, age,  general practice, diagnosis date | Schlienger et al^36^ |
| Adjusted OR, 0.90 (0.82 – 0.98) | 30 day mortality | Conditional logistic regression | Statin users: 1120319  Statin nonusers: 1120319 | Kwong et al^37^ |
| Adjusted OR,  Moderate Dose, 0.49 (0.26 – 0.76) | In hospital mortality | Logistic regression | Statin users: 19058,  Low Dose – 7475,  Moderate Dose - 11583  Statin nonusers: 57174 | Frost et al^38^  (Matched Cohort) |
| Adjusted OR,  Moderate dose, 0.62 (0.43 – 0.91) | In hospital mortality due to influenza/pneumonia | Logistic regression | Statin user cases: 31 of 397  Statin user controls: 3547 of 54136 | Frost et al^38^  (Case-Control Study) |
| Adjusted OR, 0.54 (0.42 – 0.70) | 30 day mortality | Logistic regression and  propensity score | Statin users: 1567  Statin nonusers: 4924 | Mortensen et al^39^ |
| Adjusted OR, 0.36 (0.14 – 0.92) | 30 day mortality | Multivariable logistic egression and propensity score | Statin users: 110  Statin nonusers: 677 | Mortensen et al ^40^ |
| Adjusted OR,  30 day mortality; 0.69 (0.58 – 0.82) | 30 day mortality | Logistic regression and  propensity score | Statin users: 1372  Statin nonusers: 28528 | Thomsen et al^41^ |
| Adjusted OR, 1.12 (0.77 – 1.64 | In hospital mortality or  admission to ICU | Logistic regression | Statin users: 325  Statin nonusers: 3090 | Majumdar et al ^42^ |
| Adjusted OR,  30 day mortality, 0.46 (0.25-0.85) | 30 day mortality | Logistic regression | Statin users: 257  Statin nonusers: 750 | Chalmers et al ^43^ |
| Adjusted OR, 0.33 (0.19 – 0.58) | 30 day mortality | Cox regression | Statin users: 357  Statin nonusers: 3353 | Myles et al ^44^ |
| Adjusted OR, 0.67 (0.49 – 0.91) | 6 months all cause mortality | Cox regression | Statin users: 7675  Statin nonusers: 1398 | Douglas I et al ^45^ |
| Adjusted OR, 0.90 (0.63-1.29) | 90 day mortality | Forward stepwise regression, propensity score | Statin users: 426  Statin nonusers: 1469 | Yende S et al ^46^ |
| Adjusted OR, 0.85 (0.79-0.90 | In hospital mortality | Multivariable logistic regression and propensity score | Statin users: 23285  Statin nonusers: 97969 | Rothberg MB et al ^47^ |
