# Supplementary material for: The Role of Statins in Prevention and Treatment of Community Acquired Pneumonia: A Systematic Review and Meta-Analysis
Source: PLoS One. 2013 Jan 7;8(1):e52929. doi: 10.1371/journal.pone.0052929 (PMC3538683; doi:10.1371/journal.pone.0052929)
Supplement: Table S5 — Confounders Adjusted for in Statin Treatment Group. (DOC) [file pone.0052929.s006.doc]

| **Table S5. Confounders Adjusted for in Statin Treatment Group** | | | | | | |  |
| --- | --- | --- | --- | --- | --- | --- | --- |
| **Source** | **Demographics** | **Comorbidities^1^** | **Prior Vaccination** | **Smoking** | **Severity of Illness** | **Concurrent Medications^3^** | **Propensity Scores** |
| Schlienger et al^36^ | *+* | *9* | *-** | *+* |  | *2* | *-* |
| Kwong et al^37^ | *+* | *7* | *-* | *-* | *-* | *-* | *-* |
| Frost et al^38^ | *+* | *CI* | *+* | *-* | *-* | *-* | *-* |
| Mortensen et al^39^ | *+* | *8* | *-* | *+ ;Tobacco use* | *PSI* | *-* | *+* |
| Mortensen et al ^40^ | *+* | *CI* | *-* | *-* | *-* | *3* | *+* |
| Thomsen et al^41^ | *+* | *CI* | *-* | *-* | *-* | *5* | *+* |
| Majumdar et al ^42^ | *+* | *8* | *+* | *+* | *PSI* | *5* | *-* |
| Chalmers et al ^43^ | *+* | *5* | *-* | *+* | *PSI, CURB-65* | *0* | *-* |
| Myles et al ^44^ | *+* | *CI, TI* | *-* | *+* | *-* | *3* | *-* |
| Douglas I et al ^45^ | *+* | *15* | *-* | *+* | *-* | *15^&^* | *-* |
| Yende S et al ^46^ | *+* | *CI,10,* | *+* | *+* | *PSI, APACHE III, SOFA* | *3* | *+* |
| Rothberg MB et al ^47^ | *+* | *12* | *-* | *+* | *-* | *6* | *+* |

*^1^, Number of comorbidities and risk factors defined in the article (Some comorbidities which were not found to be significant in the specific study have not been listed); ^2^, The number of medications for which adjustment was required as they were significant)*

*+, The analysis was adjusted for this variable; -, The analysis was not adjusted for this variable*

*CI, Charlson’s Comorbidity index;.PSI, Pneumonia severity index;* *APACHE, Acute Physiology and Chronic Health Evaluation; SOFA, sequential organ failure assessment*

**, Excluded as they were not significant in the univariate analysis.*

*^&^ After Propensity matching*

*Note – There may have been more than the enumerated comorbidities in the studies but only those for which adjustment performed is included*
