# Supplementary material for: The Role of Statins in Prevention and Treatment of Community Acquired Pneumonia: A Systematic Review and Meta-Analysis
Source: PLoS One. 2013 Jan 7;8(1):e52929. doi: 10.1371/journal.pone.0052929 (PMC3538683; doi:10.1371/journal.pone.0052929)
Supplement: Table S6 — Modified Newcastle-Ottawa Quality Assessment Scale for Cohort Studies included in the Meta-analysis. The criteria used for selection, comparability and outcome were - Selection was based on representativeness of the exposed cohort, selection of the non-exposed cohort, and ascertainment of exposure; Comparability of cohorts was on the basis of the design or analysis; Outcome assessment was based on the follow up and its adequacy. (DOC) [file pone.0052929.s007.doc]

| **Table S6: Modified Newcastle-Ottawa Quality Assessment Scale for Cohort Studies included in the Meta-analysis** | | | | | | | | |
| --- | --- | --- | --- | --- | --- | --- | --- | --- |
| **Included Studies** | **Selection*** | | | | **Comparability^•^** | **Outcome⁰** | | |
|  | **Representativeness of the Exposed Cohort** | **Selection of the Non-exposed Cohort** | **Ascertainment of Exposure** | **Incident Disease** |  | **Assessment of outcome** | **Length of follow-up** | **Adequacy of follow-up** |
| Smeeth et al^31^ | D | A | A | A | A | B | A | A |
| Fleming et al^32^ | A | A | A | A | C | B | NR | NR |
| Kwong et al^37^ | A | A | A | A | A | B | A | A |
| Frost et al^38^ | A | A | A | A | A | B | A | A |
| Mortensen et al^39^ | A | A | A | A | A | B | A | A |
| Mortensen et al ^40^ | A | A | A | A | A | B | A | A |
| Thomsen et al^41^ | B | A | A | A | A | B | A | A |
| Majumdar et al ^42^ | A | A | A | A | A | A | NR | NR |
| Chalmers et al ^43^ | A | A | A | A | A | D | A | C |
| Myles et al ^44^ | A | A | A | A | A | B | A | A |
| Douglas I et al ^45^ | B | A | A | A | A | B | A | A |
| Yende S et al ^46^ | A | A | A | A | A | B | A | A |
| Rothberg MB et al ^47^ | A | A | A | A | A | B | A | A |

****Selection:***

*(1) Representativeness of the exposed cohort: A, truly representative of the average patient with community-acquired pneumonia; B, somewhat representative of the average patient with community acquired pneumonia; C, selected group; D, no description of the derivation of the cohort*

*(2) Selection of the non-exposed cohort: A, drawn from the same community as the exposed cohort; B, drawn from a different source; C, no description of the derivation of the non exposed cohort.*

*(3) Ascertainment of exposure: A, secure record (e.g., surgical record); B, structured interview; C, written self-report; D, no description.*

*(4) For demonstration that the outcome of interest was not present at start of study: A, yes; B, no.*

***^•^Comparability:*** *For comparability of cohorts on the basis of the design or analysis:*

*A, study controls for co-morbidities; B, study controls for any additional factor (e.g., age and severity of illness); C, not done.*

***⁰Outcome:***

*(1) Assessment of outcome: A, independent blind assessment; B, record linkage; C, self-report; D, no description.*

*(2) Was follow-up long enough for outcomes to occur? A, yes (i.e. in-hospital or up to 30 days); B, no.*

*(3) Adequacy of follow-up of cohorts: A, complete follow-up and all subjects accounted for; B, subjects lost to follow-up was unlikely to introduce bias, because a small number were lost (i.e., 190% were available for follow-up) or a description was provided of those lost; C, follow-up rate 90% or lower (select an adequate percentage) and no description of those lost; D, no statement; NR, not reported*
