# Supplementary material for: The Role of Statins in Prevention and Treatment of Community Acquired Pneumonia: A Systematic Review and Meta-Analysis
Source: PLoS One. 2013 Jan 7;8(1):e52929. doi: 10.1371/journal.pone.0052929 (PMC3538683; doi:10.1371/journal.pone.0052929)
Supplement: Table S7 — Modified Newcastle-Ottawa Quality Assessment Scale for Case-Control Studies included in the Meta-analysis. The criteria used for selection, comparability and outcome were - Selection was based on case definition, representativeness of the cases, selection and definition of controls; Comparability of cases and controls was on the basis of the design or analysis; Outcome was based on ascertainment of exposure of both cases and controls. (DOC) [file pone.0052929.s008.doc]

| **Table S7: Modified Newcastle-Ottawa Quality Assessment Scale for Case-Control Studies included in the Meta-analysis** | | | | | | | | |
| --- | --- | --- | --- | --- | --- | --- | --- | --- |
| **Included Studies** | **Selection*** | | | | **Comparability^•^** | **Outcome⁰** | | |
|  | **Adequacy of**  **Case Definition** | **Representativeness of the Cases** | **Selection of Controls** | **Definition**  **of**  **Controls** |  | **Ascertainment**  **of**  **Outcome** | **Same Method of**  **Ascertainment for**  **Cases and Controls** | **Non-Response**  **Rate** |
| Dublin et al ^30^ | A | A | A | A | A | A | A | NR |
| Myles et al^33^ | A | B | A | B | A | A | A | NR |
| Van De Garde et al ^34^ | A | A | A | A | A | A | A | NR |
| Vinogradova et al ^35^ | A | A | A | A | A | A | A | NR |
| Schlienger et al^36^ | A | A | A | A | A | A | A | NR |
| Frost et al^38^ | B | A | B | A | A | A | A | NR |

***Selection:**

(1)Is this case definition adequate? A, yes, with independent validation; B, yes, eg record linkage or based on self reports C, no description

(2) Representativeness of the cases: A, Consecutive or obviously representative series of cases; B, Potential for selection biases or not stated

(3) Selection of controls: A, Community controls; B, Hospital controls; C, No description

(4) Definition of controls: A, No history of disease; B, No description of source

**^•^Comparability:** Comparability of cases and controls on the basis of the design or analysis:

A, study controls for co-morbidities;

B, study controls for any additional factor (e.g., age and severity of illness)

**⁰** **Outcome**

(1)Ascertainment of exposure: A, Secured records; B, Structured interview where blind to case/control status;

C, Interview not blinded to case/control status; D, written self report or medical record only (2) Same method of ascertainment for cases and controls; A, yes; B, no.

(3) Non-response rate: A, Same for both groups; B, Non-respondents described; C, Rate different and no designation; NR, not reported
