# Supplementary material for: The Role of Statins in Prevention and Treatment of Community Acquired Pneumonia: A Systematic Review and Meta-Analysis
Source: PLoS One. 2013 Jan 7;8(1):e52929. doi: 10.1371/journal.pone.0052929 (PMC3538683; doi:10.1371/journal.pone.0052929)
Supplement: Supplementary file 9 [file pone.0052929.s001.doc]

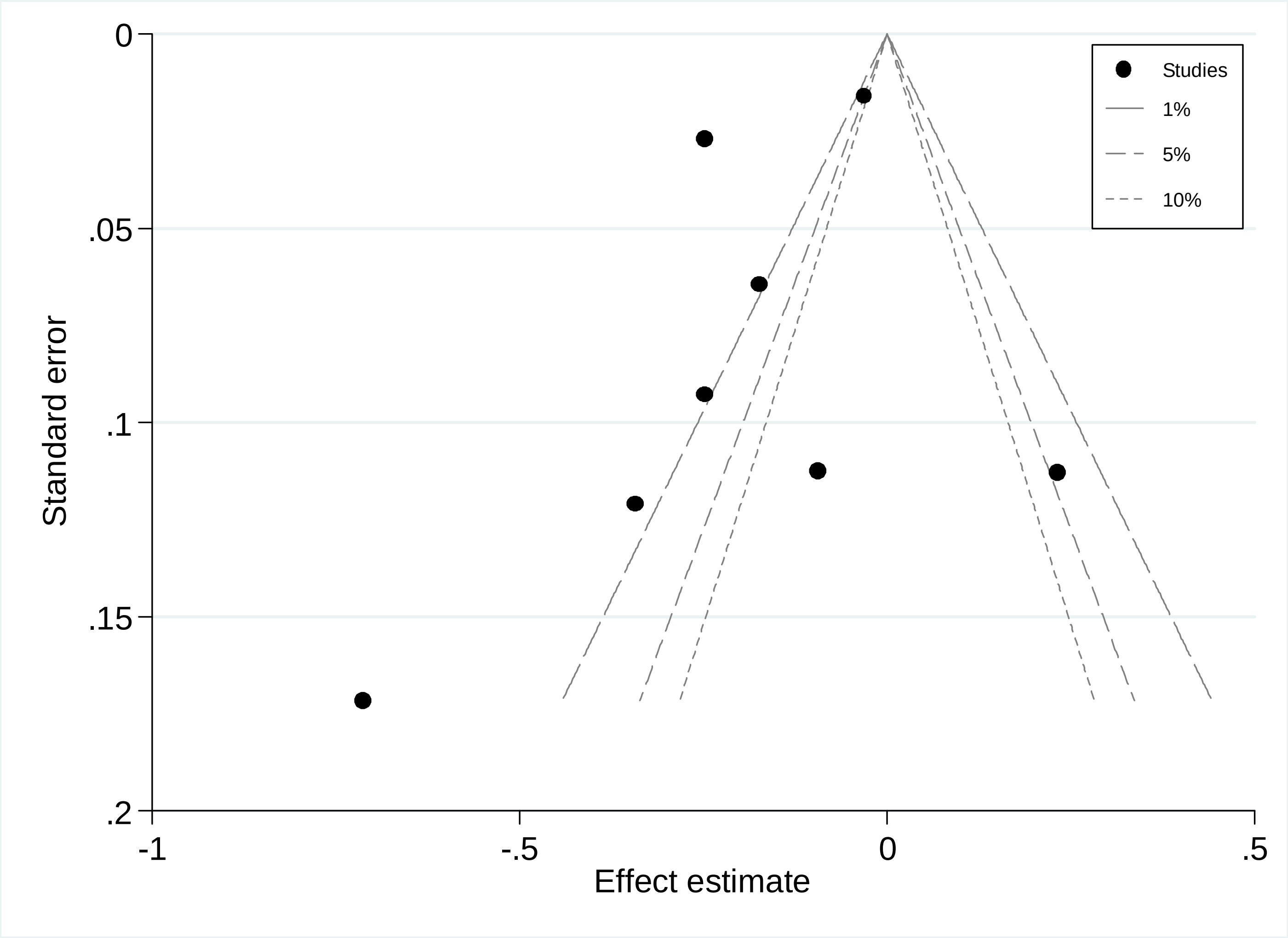
Studies
1%
5%
10%
0
.05
.1
.15
.2
Standard error
-1
-.5
0
.5
Effect estimate
